# Notes and Formulæ

**Published:** 1889-03

**Authors:** 


### PRACTICAL NOTES AND FORMULÆ.

---

**Ointment for Hemorrhoids.**—

| | | | |
|---|---|---|---|
| ℞ | Ungent. populi - - - | 30 | grams, |
| | Cerat. saturni - - . | 10 | " |
| | Ext. belladonnæ - - - | | " |
| | Ext. opii - - - - | ää 1 | " |
| | Antipyrine - - - | 3 | " |

M.—Apply to the part as needed. It calms itching and stops bleeding in phthisis:

| | | | |
|---|---|---|---|
| ℞ | Tannin (alcohol) - - - | 5 | grams. |
| | Glycerine - - - - - | 30 | " |
| | Sherry wine - - - | 1000 | " |

M. This makes a part of wine, which has been highly recommended in consumption. It is given after meals.—*Med. Times.*

**Seborrhœa.**—In his Atlas of Venereal and Skin Diseases, Dr. P. A. Morrow recommends the following treatment for seborrhœa of the scalp: First, loosen all scalps; then shampoo with spiritus saponis kalinus and warm water, and dry. After this apply the following ointment: ℞ Acidi tannici, ʒi; glycerinii puri, ʒi; petrolati, ʒii; ung. aquæ rosæ, ℥j. M. ft. ung. To prevent the reformation of crusts apply: ℞ Sulfuris loti, ʒi; adipis, ℥i. M.

**Menorrhagia.**—℞ Acid. gallici glycerini, P. B., ℥j; acid. sulph. dil., ʒij; ext. ergotæ fluidæ ʒiij; aq. cinnamoni ad, ℥viij. One-eighth part every four hours, in profuse discharge.—*Med. Digest.*

**Amenorrhea.**—Prof. Parvin prescribes the following in some cases of amenorrhea in anemic subjects, and the result in many cases has been gratifying: ℞ Feiri sulph. ex.; terebinth. albæ; pulv. aloes, ää gr. j. M. Ft. pil. I. Sig. One t. d. The quantity of aloes may have to be reduced.—*Med. Digest.*

**Ringworm.**—

| | | |
|---|---|---|
| ℞ | Acid chrysophanci . . . . . . | dr. j. |
| | Lanolin . . . . . . . . | oz. j. |

Ft. unguent.—*Med. World.*

**Epilation, Removal of Crusts, etc.**—

| | | |
|---|---|---|
| ℞ | Hydrargyri ammoniati, | |
| | Fror. sulphuris . . . . . . | ää dr. j |
| | Vaselini . . . . . . . | ad. oz. j |

Ft. urguent.—*Ib.*

| | | |
|---|---|---|
| ℞ | Tinct. Aconiti, | |
| | Tinct. belladonnæ . . . . . | ää m xjv |
| | Aq. camph. . . . . . . . | oz. ij |

One teaspoonful every hour at commencement.—*Ib.*

| | | |
|---|---|---|
| ℞ | Potass. bitartratis . . . . . . | ℈ j |
| | Vin. ipecacuanhæ . . . . . . | m v |
| | Sp. etheris nit. . . . . . . | m xv |
| | Inf. digitalis . . . . . . . | m xl |
| | Aq. dest. . . . . . . . | ad. oz. ss |

Three times a day, for children over five years, in scarlatenal nephritis.—*Ib.*

**To Prevent Scarlet Fever.**—

| | | |
|---|---|---|
| ℞ | Tinct. belladonnæ . . . . . . | m l |
| | Aq. dest. . . . . . . . . . | oz. ij |

One teaspoonful four times a day, as a prophylactic when scarlet fever is in the house.—*Ib.*

**A Hair Restorer.**—Make a pommade with three ounces of lanolin and five drachms of lard, and a little essence of roses. Shake up seventy-five grains of subnitrate of bismuth, and forty-five grains of citric acid in three and a half drachms of glycerine, and add the mixture to the pommade. This is stated to sestore white or gray hair to its pristine brown color.

**For the Constipation of Children** where the stools are clay colored and hard, for a child of one Aear old, Dr. Rex recommends:

| | | |
|---|---|---|
| ℞ | Podophyllin . . . . . . . . | gr. $\frac{1}{20}$, |
| | Spirit. vini rectificat . . . . . | m xx |
| | Syrup . . . . . . . . . . | fl. oz. j |

M. Sig.—fl. dr. j ter die.

